# Ciliogenesis and Hedgehog signalling are suppressed downstream of KRAS during acinar-ductal metaplasia in mouse

**DOI:** 10.1242/dmm.044289

**Published:** 2020-07-30

**Authors:** Fiona K. Bangs, Paul Miller, Eric O'Neill

**Affiliations:** 1 Department of Oncology, Medical Sciences Division, University of Oxford, Old Road Campus Research Building, Headington, Oxford OX3 7DQ, UK; 2 Ludwig Institute for Cancer Research, Nuffield Department of Medicine, University of Oxford, Old Road Campus Research Building, Headington, Oxford OX3 7DQ, UK

**Keywords:** Acinar-ductal metaplasia, Hedgehog signalling, KRAS, Pancreatic ductal adenocarcinoma, Primary cilia

## Abstract

Pancreatic ductal adenocarcinoma (PDAC) is the third leading cause of cancer-related deaths worldwide, but has a 5-year survival rate of only 7% primarily due to late diagnosis and ineffective therapies. To treat or even prevent PDAC, it is vital that we understand the initiating events that lead to tumour onset. PDAC develops from preneoplastic lesions, most commonly pancreatic intraepithelial neoplasias (PanINs), driven by constitutive activation of KRAS. In patients, PanINs are associated with regions of acinar-to-ductal metaplasia (ADM) where, in response to inflammation, acini dedifferentiate to a pancreatic progenitor-like fate. In healthy tissue this process is reversible leading to regeneration of the pancreas; however, in the presence of oncogenic KRAS, regeneration is blocked and ADM can give rise to PanIN lesions. Here, we used a 3D mouse acinar culture that recapitulates ADM *in vitro* to explore how KRAS prevents regeneration. Regeneration is regulated by Hedgehog (Hh) signalling, which is transduced via the primary cilium. In wild-type acini, cilia assemble upon ADM and Hh target gene expression is upregulated; however, ciliogenesis and Hh signalling are suppressed during ADM in cells expressing oncogenic KRAS. We show that ciliogenesis fails due to ectopic activation of the cilium disassembly pathway, which is mediated by AurkA, a direct transcriptional target of KRAS. Inhibition of AurkA is able to rescue primary cilia and restore Hh signalling. We suggest that this could be used as a mechanism to prevent the formation of early lesions and thereby prevent progression to PDAC.

## Abstract (precis)

**Summary:** Following injury, constitutive activation of KRAS in the murine pancreas inhibits regeneration and instead promotes the formation of preneoplastic lesions by blocking Hedgehog signal transduction by forcing disassembly of the primary cilium.

## INTRODUCTION

Pancreatic ductal adenocarcinoma (PDAC) is the third leading cause of cancer-related deaths worldwide, with a 5-year survival rate of only 7% (Rahib et al., 2014; McGuigan et al., 2018; Siegel et al., 2015). This is because patients go undiagnosed until they develop advanced, often-metastatic disease, when treatment options are very limited and not curative. If we are to improve patient survival, it will be necessary to detect PDAC sooner, and to do this we need to understand the cellular and molecular events that give rise to this disease.

PDAC originates from precursor neoplastic lesions, including intraductal papillary mucinous neoplasms and mucinous cystic neoplasms, but is most commonly derived from pancreatic intraepithelial neoplasias (PanINs), which are classified as low grade to high grade with increasing cellular atypia. Low-grade PanIN lesions are associated with mutations leading to constitutive activation of KRAS, the major oncogenic driver of PDAC and considered the primary genetic event to initiate disease. However, oncogenic activation of KRAS alone is not sufficient to drive tumour initiation; mouse models of PDAC in which KRAS is activated in the acinar compartment have shown that chronic inflammation is also required (Guerra et al., 2007). Inflammation in the pancreas leads to acinar-to-ductal metaplasia (ADM), a normal homeostatic process by which acini dedifferentiate, downregulating acinar-specific genes such as amylase and *Mist1* (also known as *Bhlha15*) and upregulating pancreatic progenitor genes including nestin, *Hes1*, *Pdx1* and *Sox9* (Miyatsuka et al., 2006; Morris et al., 2010; Song et al., 1999). In a healthy pancreas, these progenitor-like cells act as facultative stem cells to regenerate the exocrine pancreas (Desai et al., 2007; Shi et al., 2013; Jensen et al., 2005; Means et al., 2005); however, following constitutive activation of KRAS, these progenitor-like cells fail to redifferentiate. Instead, signalling downstream of KRAS maintains progenitor gene expression (Morris et al., 2010; Guerra et al., 2007; Carrière et al., 2009), promoting the development of PanINs, and in patients, ADM is observed in proximity to high-grade PanINs (Liou et al., 2015; Collins et al., 2014; Brune et al., 2006; Habbe et al., 2008; Grippo et al., 2003; de La O et al., 2008; Parsa et al., 1985).

Under healthy circumstances, regeneration is driven by Hedgehog (Hh) signalling, which is upregulated upon pancreatic injury (Thayer et al., 2003; Kayed et al., 2006). Inhibition of Hh signalling prevents redifferentiation, resulting in persistent progenitor-like cells and failure to regenerate akin to that seen following constitutive activation of KRAS^G12D^ (Fendrich et al., 2008). In vertebrates, Hh signalling is transduced via the primary cilium, a non-motile microtubule-based organelle that projects from the cell surface (Bangs and Anderson, 2016). Most vertebrate cells have a single primary cilium including pancreatic ductal cells, centroacinar cells, and alpha, beta and delta cells within the islets of Langerhans (Lodh et al., 2014). Acini, however, are not ciliated, yet upon ADM a cilium is assembled (Seeley et al., 2009), consistent with a requirement of Hh signalling for redifferentiation of progenitor-like cells. As such, genetic deletion of cilia in the pancreas prevents regeneration akin to Hh inhibition, leading to persistent pancreatitis and eventually cystogenesis (Cano et al., 2004).

Primary cilia are dynamic structures that assemble and disassemble in synchrony with the cell cycle. During interphase, the cilium is assembled from the distal end of the mother centriole, which matures to form a basal body acquiring appendage proteins that dock it to the plasma membrane. Microtubules then extend into the plasma membrane forming the ciliary axoneme via a process called intraflagellar transport (IFT). Prior to mitosis, the cilium is disassembled to release the mother centriole, which is required for efficient organisation of the spindle pole and cytokinesis. Cilium disassembly is regulated by the mitotic kinase, AurkA, which promotes axonemal microtubule depolymerization through activation of histone deacetylase 6 (HDAC6) (Pugacheva et al., 2007).

To further understand how KRAS prevents regeneration and promotes PanIN formation, we assessed Hh signalling and the status of primary cilia during ADM in mice expressing constitutively active KRAS^G12D^ under the control of a pancreas-specific Pdx1Cre, LSL-KRAS^G12D^;Pdx1Cre (Hingorani et al., 2003), referred to as KC. We show that, upon ADM, KC cells assemble fewer cilia compared to wild type (WT) and this renders them less responsive to Hh signals. We demonstrate that cilia loss is not due to immature basal bodies or absence of IFT machinery but through ectopic activation of the cilium disassembly pathway. Through inhibition of AurkA, we are able to rescue cilia, restore the response to Hh signalling and promote redifferentiation.

## RESULTS

### Primary cilia assemble upon ADM *in vivo* and *in vitro*

**Fig. 1.**
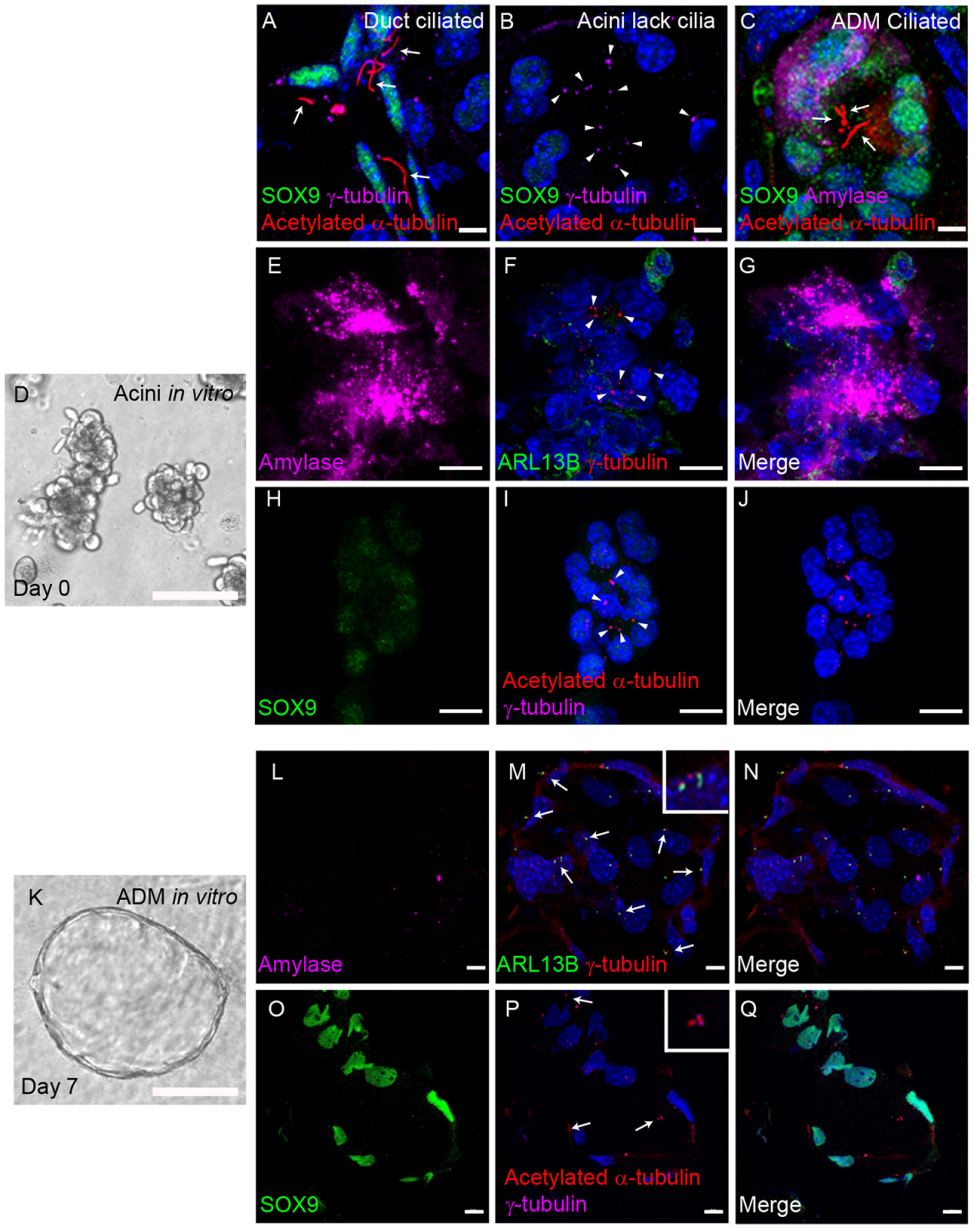
**Cilia are acquired upon acinar-to-ductal metaplasia *in vivo* and *in vitro*.** (A) In regions of healthy pancreas from KC mice, primary cilia marked with acetylated α-tubulin (red, arrows) are present on ductal cells labelled with SOX9 (green). (B) Acini have clearly visible centrosomes marked with γ-tubulin (magenta, arrowheads) but lack primary cilia. (C) Upon ADM in KC mice, acini marked with amylase (magenta) upregulate the ductal marker SOX9 (green) and assemble primary cilia marked with acetylated α-tubulin (red, arrows). (D) Brightfield image of acini *in vitro* immediately following plating. (E-J) Day 0 ADM assay: acini express amylase (E, magenta) but not SOX9 (H, green) and lack primary cilia marked with ARL13B (F, green) or acetylated α-tubulin (I, red), although centrosomes are present marked with γ-tubulin (F, red; I, magenta, arrowheads). (K) Brightfield image of ductal metaplasia 7 days after plating. (L-Q) Day 7 ADM assay: ductal cysts no longer express amylase (L, magenta) and upregulate SOX9 (P, green). Primary cilia are present marked with ARL13B (M, green, arrows) or acetylated α-tubulin (P, red, arrows), projecting from basal bodies marked with γ-tubulin (M, red; P, magenta). Nuclei are stained with DAPI (blue), Scale bars: 10 µm (A-C,E-J,L-Q), 50 µm (D,K).

Most vertebrate cells are ciliated, including ductal and centroacinar cells of the pancreas, which display a ciliary axoneme labelled with acetylated α-tubulin projecting from the basal body labelled with γ-tubulin (Fig. 1A). Acini, however, are one of the few cells types (along with haematopoietic cells and hepatocytes) that lack cilia. In these cells, centrosomes are clearly visible yet no ciliary axoneme is detected (Fig. 1B). In response to injury, acini undergo ADM, during which amylase is downregulated and ductal markers such as SOX9 are expressed. Concurrent expression of both markers is used to identify ADM cells, and we and others observe that cilia assemble in these cells (Fig. 1C; Seeley et al., 2009).

It is possible to mimic ADM *in vitro*, where – over a period of 7 days – clusters of acini cultured in 3D undergo metaplastic transformation, forming ductal cysts (Fig. 1D-Q). As *in vivo*, acini on the first day of culture express amylase, very low levels of SOX9 and lack cilia, despite clearly visible centrosomes (Fig. 1D-J). Whereas amylase is downregulated, following 7 days in culture, SOX9 is upregulated and cilia are present, marked with both acetylated α-tubulin and cilia-specific marker ARL13B (Fig. 1K-Q). Thus, cilia assembly following ADM is recapitulated *in vitro* as observed *in vivo*.

### Constitutive activation of KRAS promotes ADM and blocks ciliogenesis

More than 90% of PDAC cases are associated with mutations in KRAS, commonly a glycine to aspartic acid at position 12 that reduces its affinity for GTPase-activating proteins, thereby locking KRAS in a constitutively active state. These mutations have been detected in low-grade PanIN lesions and are therefore considered the primary initiating event leading to PDAC. In mice, expression of an inducible KRAS^G12D^ in the pancreas using LSL-KRAS^G12D^;Pdx1Cre (Hingorani et al., 2003) (KC) recapitulates the human disease, giving rise to precursor PanIN lesions and, in ∼7% of cases, forming metastatic PDAC by 6-9 months.

**Fig. 2.**
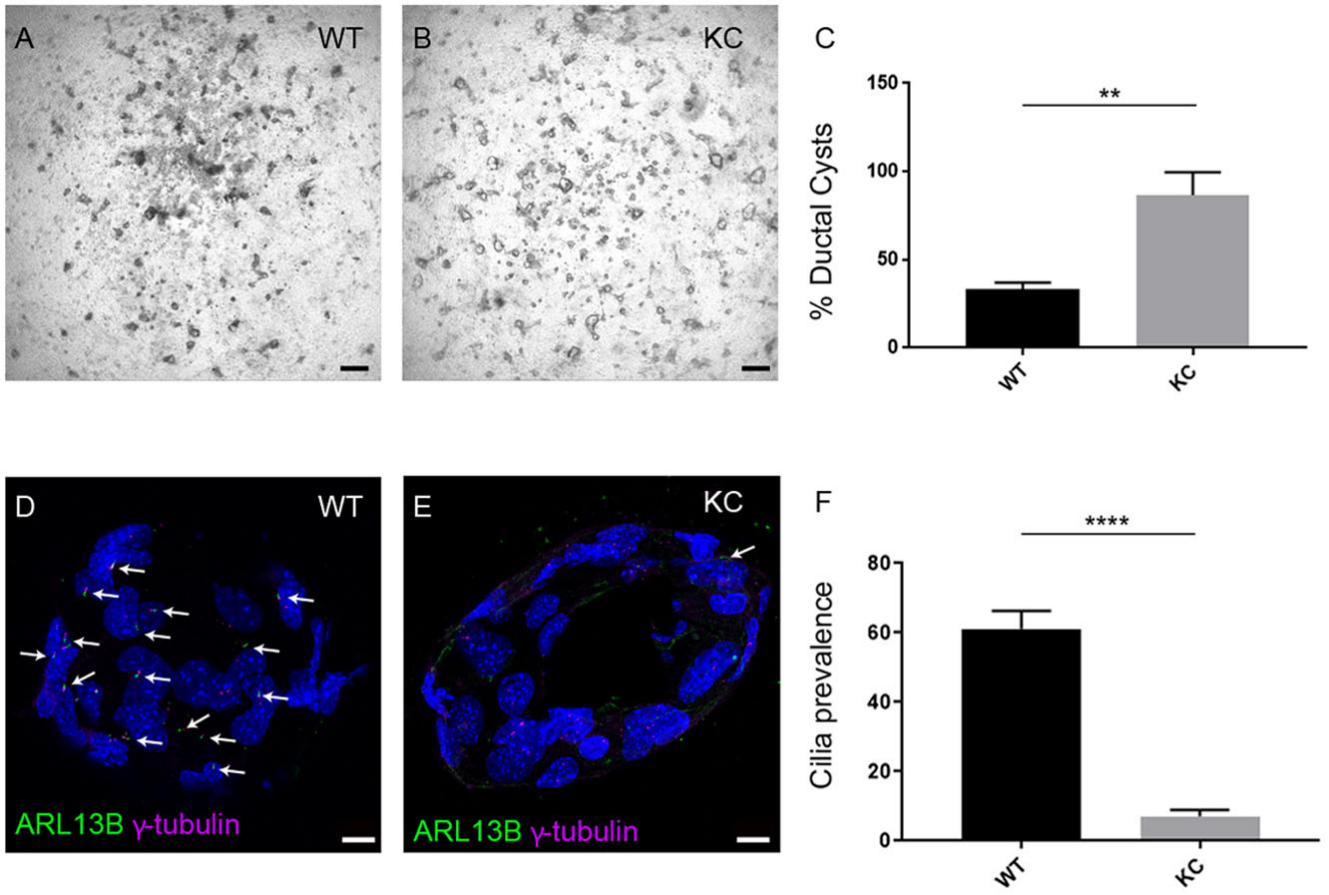
**Constitutive activation of KRAS promotes ADM but is associated with fewer primary cilia.** (A,B) Brightfield images of representative ADM assays showing more ductal structures in KC compared to WT, quantified in C. (C) Percentage of ductal cysts formed by day 7: WT 33±4%, KC 87±13% (*n*=3 biological replicates). (D,E) Primary cilia marked with ARL13B (green, arrows) are more prevalent on WT ADM (D) compared to KC (E). Basal bodies are marked with γ-tubulin (magenta), nuclei are stained with DAPI (blue). (F) Quantification of cilia percentage: 61±5.3% cells ciliated in WT (*n*=266 cells from two biological replicates) compared to 7.2±1.8 in KC ADM (*n*= 297 cells from two biological replicates). Data are mean±s.e.m. **P<0.01, ****P<0.0001 (two-way ANOVA). Scale bars: 100 µm (A,B), 10 µm (D,E).

As shown previously, acini from KC mice have a higher propensity to undergo ADM *in vitro*, where an average of 87±13% acini form ductal cysts compared to 33±4% in WT controls [*P*=0.0059, *n*=3; Fig. 2A-C (Shi et al., 2013)]. Hh signalling is required for redifferentiation, and primary cilia are essential for the transduction of Hh signals (Fendrich et al., 2008; Bangs and Anderson, 2016). Specifically, they are required to activate GLI-Kruppel family members, the transcriptional effector of the Hh pathway in response to ligand, leading to expression of Hh target genes, including the Hh receptor patched 1 (*Ptch1*) and *Gli1*. As KC cells have a higher propensity to undergo ADM, we explored whether this is due to an inability to respond to Hh signalling by analysing primary cilia presence and function in these cells.

Cilia prevalence was calculated as a percentage of interphase cells [identified as those without condensed chromosomes using 4′,6-diamidino-2-phenylindole (DAPI)]. Immunofluorescence staining of sections taken through ductal cysts revealed that primary cilia assemble; in KC ductal cysts, however, there are fewer cilia, 7.2±1.8% compared to 61±5.3% in WT (*P*<0.0001), even though the basal body is clearly present, marked with γ-tubulin (Fig. 2D-F; *n*=12 ducts from two biological repeats). This is consistent with reduced primary cilia in PDAC cell lines (Nielsen et al., 2008), and it has been shown that this is a consequence of oncogenic KRAS signalling as KRAS knockdown significantly increased cilia formation (Kobayashi et al., 2016).

### Hh signalling is downregulated in ductal cysts with constitutive activation of KRAS

**Fig. 3.**
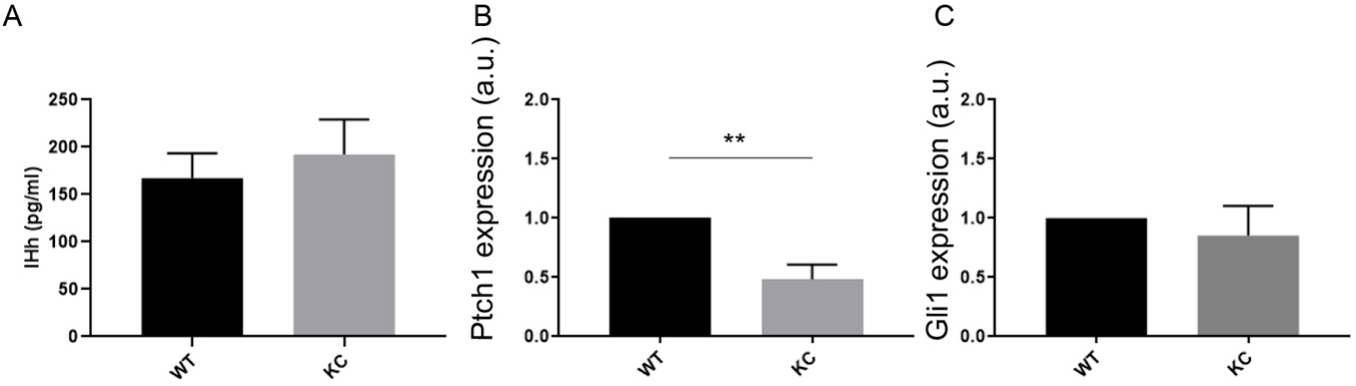
**KC ductal cysts have a reduced capacity to respond to Hh signalling.** (A) WT and KC ductal cysts secrete Ihh in similar amounts (*n*=2 biological replicates). (B) *Ptch1* expression is significantly reduced in KC compared to WT (*n*=6 biological replicates). Data are mean±s.e.m. **P<0.01 (two-way ANOVA). (C) *Gli1* expression is not significantly decreased due to non-canonical regulation via KRAS (*n*=6 biological replicates). a.u., arbitrary units.

A reduction in cilia would suggest that KC cells have a reduced capacity to respond to Hh. Upon ADM, Indian hedgehog (IHH) is expressed *in vivo* (Kayed et al., 2003) and enzyme-linked immunosorbent assays (ELISAs) show that Ihh is also secreted from both WT and KC cells undergoing ADM *in vitro* (Fig. 3A; *n*=2). We then assessed whether these cells can respond to Ihh using quantitative PCR (qPCR) to determine the expression level of Hh target genes *Ptch1* and *Gli1*. Compared to WT, there is a significant decrease in *Ptch1* expression in KC ductal cysts (Fig. 3B; *P*<0.002, *n*=6), indicating that the presence of fewer cilia in KC ductal cysts reduces the response of these cells to Hh signals. No significant decrease in *Gli1* expression was observed (Fig. 3C; *n*=7); however, this can be explained by the fact that *Gli1* transcription is maintained independent of Hh via a non-canonical route involving KRAS.

### Basal bodies are mature but fail to nucleate primary cilia in KC cells

We next examined how ciliogenesis is regulated in KC ADM cells. Ciliogenesis is tightly coupled with the cell cycle, during which the cilium is assembled during interphase and disassembled prior to mitosis (Plotnikova et al., 2008). This has led to the proposal that the cilium can act as a break on cell cycle progression and therefore cilia loss may result in uncontrolled proliferation. We do not observe a significant difference in Ki67 (also known as Mki67)-positive cells or the number of mitotic cells between WT and KC ADM cells *in vitro* (WT 3±2%, KC 3±4% Ki67-positive cells, *n*=4; or WT 1±1%, KC 0% mitotic cells determined using DAPI, *n*=16 ductal cysts for WT, *n*=17 ductal cysts for KC across four biological replicates); therefore, cilia loss is not because a higher proportion of KC ADM cells are in mitosis. This also suggests that cilia loss does not lead to increased proliferation in these cells.

**Fig. 4.**
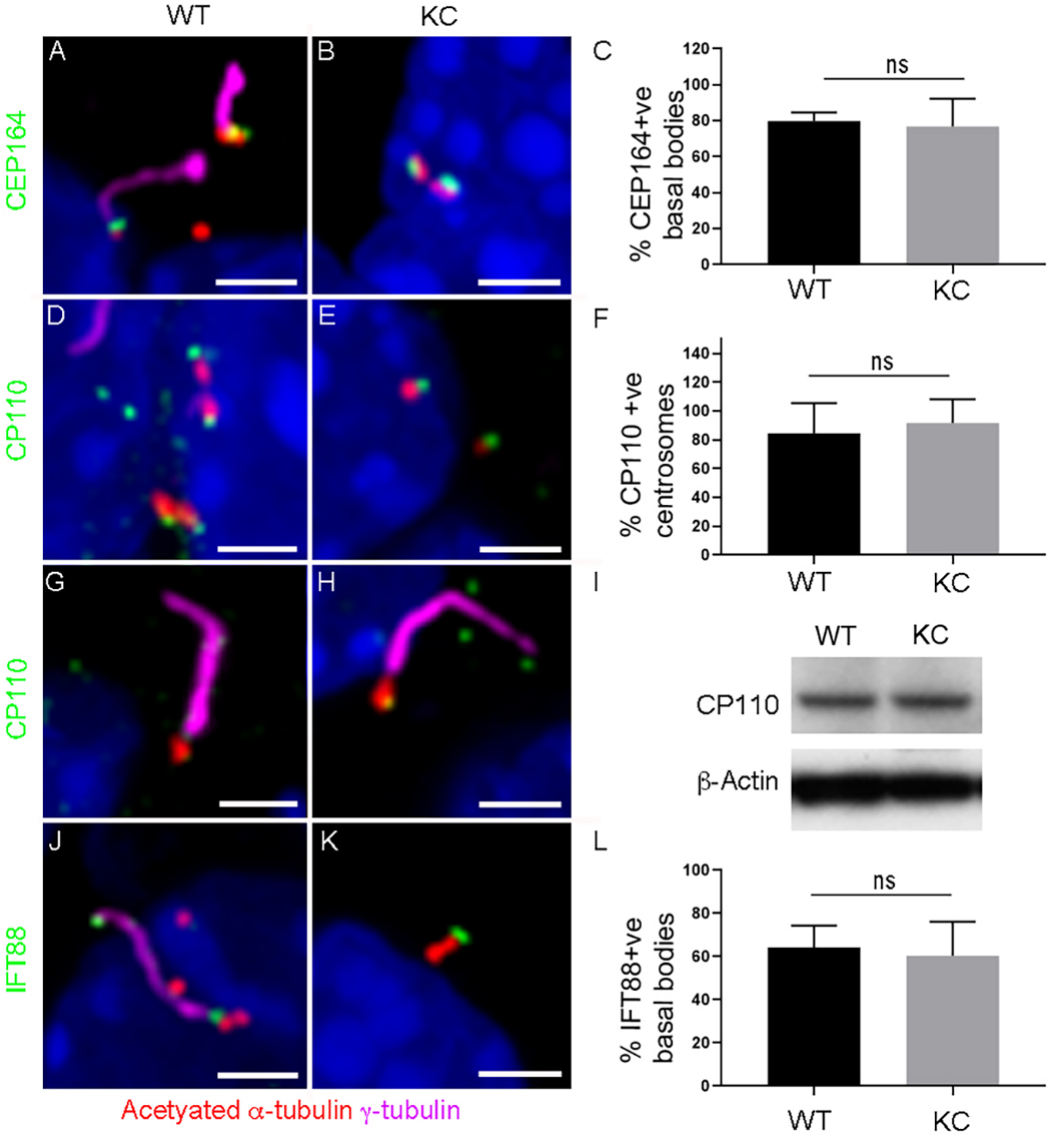
**Basal bodies are mature and associated with the IFT machinery in KC cells.** (A,B) In WT ADM cells, distal appendage protein CEP164 (green) is present at the distal end of basal bodies marked with γ-tubulin (red) in WT (A) and KC ADM cells (B). (C) Percentage of basal bodies positive for CEP164: WT 79.9±2.8%, KC 76.8±7.8% (*n*=22 basal bodies from three duct-like structures for WT and 58 basal bodies from four duct-like structures for KC, across three biological replicates). (D,E) CP110 (green) is present on centrosomes marked with γ-tubulin (red) in non-ciliated WT (C) and KC ADM cells (D). (F) Percentage of centrosomes with CP110: WT 84.7±10.5%, KC 91.7±8.4% (*n*=43 centrosomes from four duct-like cysts for WT and 63 centrosomes from four duct-like cysts for KC, across three biological replicates). (G,H) CP110 (green) is absent from the basal body marked with γ-tubulin (red) of ciliated WT (G) and KC ADM cells (H). CP110 can still be seen on the daughter centriole in this KC example. Axoneme marked with acetylated α-tubulin (magenta). (I) Western blot indicates that CP110 protein levels are equivalent in WT and KC ADM cells. (J) IFT88 (green) is present at the base and along the ciliary axoneme marked with acetylated α-tubulin (magenta) in WT ADM cells. (K) Despite absence of a ciliary axoneme, IFT88 is still present (green) on basal body marked with γ-tubulin (red) in KC ADM cells. (L) Percentage of basal bodies positive for IFT88: WT 64±7.1%, KC 60.4±6.5% (*n*=14 basal bodies from three duct-like for WT and 94 basal bodies from six duct-like structures for KC, across three biological replicates). Data are mean±s.e.m. ns, non-significant (two-way ANOVA). Nuclei stained with DAPI (blue). Scale bars: 2 µm.

Primary cilia assemble from the distal end of the basal body. The basal body matures from the mother centriole, acquiring distal appendage proteins that dock it to the plasma membrane, a prerequisite to axoneme extension (Tanos et al., 2013). To establish whether basal bodies mature and dock in KC ADM cells, we assessed for the presence of distal appendage protein CEP164 (Graser et al., 2007). In WT ADM cells, CEP164 is present at the junction between the basal body and ciliary axoneme in 79.9±2.8% of basal bodies (Fig. 4A,C; *n*=22 basal bodies from three duct-like structures across three biological replicates). Despite the lack of cilia, CEP164 is also present on 76.8±7.8% of basal bodies in KC ADM cells (Fig. 4B,C; *n*=58 basal bodies from four duct-like structures across three biological replicates). This indicates that basal bodies are mature as they have acquired distal appendage proteins, and we can infer that they are likely to be docked to the plasma membrane. Therefore, reduced ciliogenesis in KC ADM cells is not due to lack of mature basal bodies or a failure of basal bodies to dock to the plasma membrane.

During the docking process, subdistal appendages mediate fusion of a ciliary vesicle to the distal end of the basal body into which the microtubule axoneme extends. Centrosomal protein CP110 is required for subdistal appendage assembly and subsequent ciliary vesicle fusion, after which CP110 is removed from the distal end of the basal body (Yadav et al., 2016). In non-ciliated WT ADM cells, CP110 is present at the centrosome (84.7±10.5%; Fig. 4D,F; *n*=43 centrosomes from four duct-like cysts across three biological replicates) and absent from 100% of basal bodies once the cilium is assembled (Fig. 4G; *n*=cilia from four duct-like cysts across three biological replicates). In KC ADM cells, CP110 is also present at the centrosome (91.7±8.4%; Fig. 4E,F; *n*=63 centrosomes from four duct-like cysts across three biological replicates) and removed from 100% of basal bodies in ciliated cells (Fig. 4H; *n*=cilia from three duct-like cysts across three biological replicates). KRAS has been shown to negatively regulate CP110, albeit indirectly (Hu et al., 2015); our western blot results show that CP110 protein levels are equivalent between WT and KC ADM cells (Fig. 4I). In the absence of CP110, failure of vesicle fusion allows aberrant microtubule extension, resulting in elongated basal bodies (Schmidt et al., 2009). No elongated basal bodies were observed in KC ADM cells, indicating that CP110 is present and functioning in these cells.

Once docked, the ciliary axoneme is assembled via IFT. In WT ADM cells, the IFT-B protein, IFT88, is localized to the distal end of the basal body and along the length of the ciliary axoneme of all primary cilia (Fig. 4J; *n*=14 cilia from three duct-like cysts across three biological replicates). Only 61±5.3% of WT ADM cells are ciliated, and quantification of IFT88 showed that 64±7.1% of cells have IFT88 at the basal body corresponding to ciliated cells, whereas IFT88 is not present on centrosomes of non-ciliated WT cells (Fig. 4L; *n*=14 basal bodies from three duct-like cysts across three biological replicates). IFT88 is also present at the distal end of 60.4±6.5% of basal bodies in KC ADM cells; however, these cells fail to assemble primary cilia (Fig. 4K,L; *n*=94 basal bodies from six duct-like cysts across three biological replicates). This indicates that cilia loss in KC ADM cells is not caused by a lack of IFT machinery.

### Ectopic activation of AurkA and HDAC2 accounts for reduced cilia in KC ADM cells

**Fig. 5.**
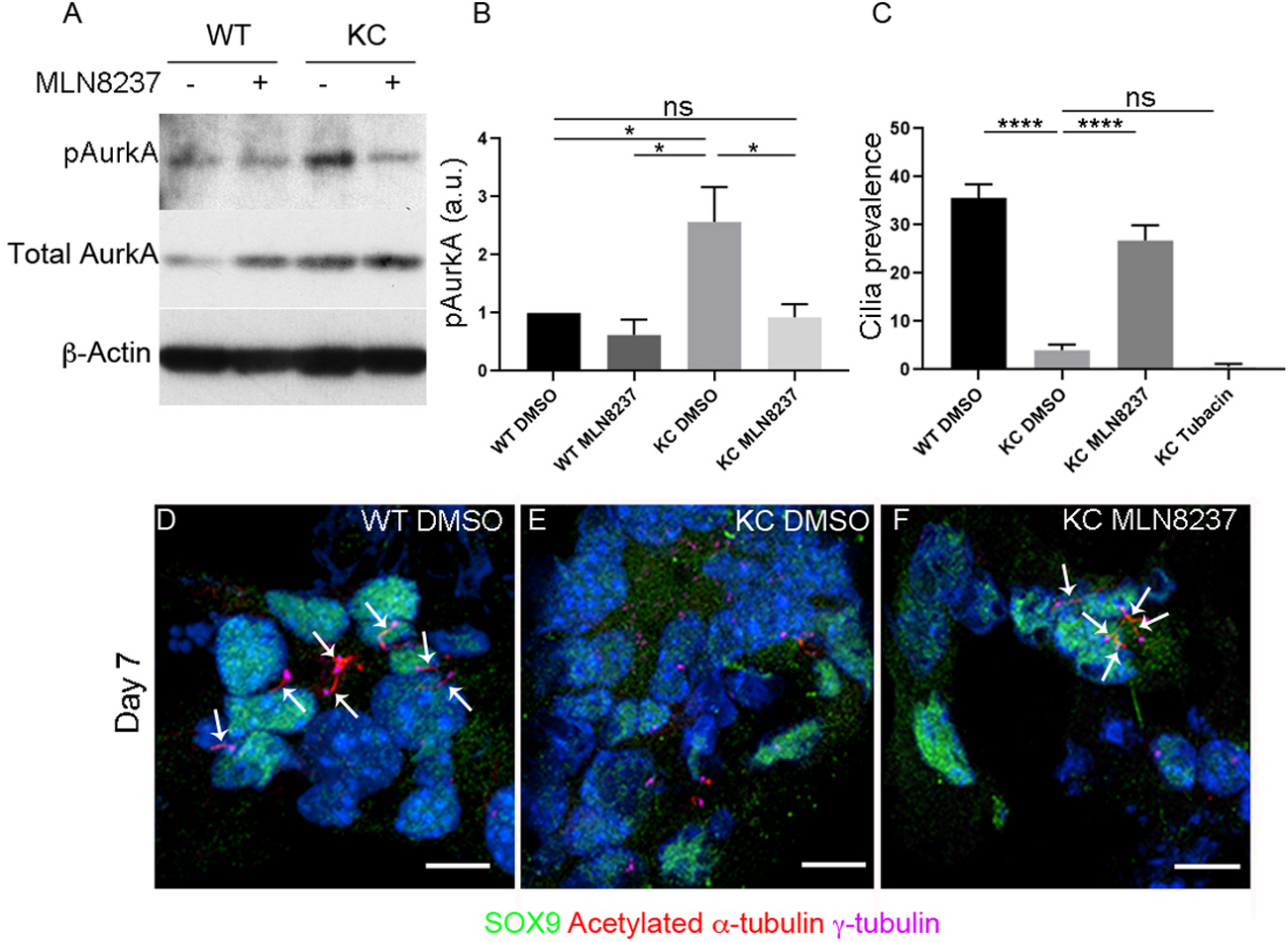
**pAurkA and HDAC2 inhibit ciliogenesis in KC ADM cells.** (A) Western blot showing that pAurkA levels are increased in KC compared to WT ADM cells, and are restored to WT levels following 7 days of treatment with 250 nm AurkA inhibitor MLN8237. (B) Quantification of western blot shown in A (*n*=4 biological replicates). (C) Cilia percentage is rescued in KC ADM following treatment with AurkA inhibitor MLN8237 but not with HDAC6 inhibitor Tubacin for 7 days. Cilia percentage in WT ADM cells: 35.65±2.8% (*n*=587 cells from four biological replicates), DMSO-treated KC ADM cells 4±1.2% (*n*=402 cells from four biological replicates), KC ADM cells treated with MLN8237 26.7±3.2% (*n*=587 cells from four biological replicates), KC ADM cells treated with Tubacin 0.62±0.62% (*n*=125 cells from one biological replicate). (D-F) Cilia marked with acetylated α-tubulin (red, arrows) are rescued in KC ADM cells following treatment with AurkA inhibitor MLN8237. Ductal metaplasia is indicated with SOX9 expression (green) and basal bodies are marked with γ-tubulin (magenta). In some cases, duct-like morphology is not discernible due to the plane of the section. Nuclei are stained with DAPI (blue). Scale bars: 10 µm. Data are mean±s.e.m. ns, non-significant; *P<0.05, ****P<0.0001 (two-way ANOVA).

Cilia are assembled during interphase but prior to mitosis the cilium is disassembled, which is thought to be necessary to allow the centrosome to coordinate efficient nucleation of the spindle pole. AurkA is classically known for its role in maintaining the centrosome and mitotic spindle prior to mitosis, but, more recently, non-mitotic roles for this kinase have been identified; namely, promoting axoneme disassembly prior to mitosis (Pugacheva et al., 2007). In non-ciliated cells such as mouse extraembryonic endodermal cells, ciliogenesis is blocked, owing to a highly active cilium disassembly pathway mediated by AurkA (Bangs et al., 2015). We next assessed whether reduced ciliation in KC ADM cells was also due to the cilium disassembly pathway. Indeed, AurkA is regulated by KRAS via the MAPK signalling pathway, and, consequently, AurkA is ectopically expressed in pancreatic cancer and KRAS knockdown in PDAC cell lines leads to depletion of AurkA and cilia formation (Furukawa et al., 2006; Li et al., 2003; Kobayashi et al., 2016). Western blotting shows that levels of phosphorylated AurkA are 2.6 times higher in KC ADM cells compared to WT (Fig. 5A,B; *P*=0.04, *n*=4), and total AurkA is increased in KC compared to WT (Fig. 5A; *P*=0.0026, *n*=4).

To ascertain whether ectopic AurkA activity can account for reduced cilia in KC ADM cells, we inhibited AurkA using a specific inhibitor, MLN8237, and assessed cilia percentage. Treatment of acinar cultures with 250 nM MLN8237 for 7 days significantly reduced the levels of phosphorylated AurkA (pAurkA) in KC ADM cells compared to dimethyl sulfoxide (DMSO)-treated controls (Fig. 5B; *n*=4, *P*=0.0288). This resulted in a significant increase in cilia percentage on duct-like cysts marked with SOX9, from 4±1.2% (*n*=402 cells from four biological replicates) in DMSO-treated KC ADM cells to 26.7±3.2% (*n*=587 cells from four biological replicates, *P*<0.0001) in MLN8237-treated KC ADM cells cultured for 7 days (Fig. 5C-F). Proliferation, which is already very low in KC ADM cells, is not further decreased upon AurkA inhibition.

One mechanism through which AurkA mediates cilium disassembly is activation of the histone deacetylase HDAC6. To ascertain whether cilium disassembly is mediated via this mechanism in KC ADM cells, we treated KC acini cultures with 5 µm Tubacin, an HDAC6-specific inhibitor, for 7 days. This treatment did not rescue cilia in KC ADM cells, in which cilia percentage was reduced further than in DMSO-treated controls – from 4±1.2% (*n*=402 cells from four biological replicates) to 0.62±0.62% (*n*=125 cells from one biological replicate). This is in line with a previous report which also showed that inhibition of HDAC6 fails to rescue primary cilia in PDAC cell lines (Kobayashi et al., 2016). This suggests that a novel mechanism downstream of AurkA mediates cilium disassembly in these cells.

### Rescue of cilia in KC ADM cells increases their response to Hh signalling and reduces their capacity to form ductal cysts in an ADM assay

Hh signalling is necessary for redifferentiation of metaplastic cells in the pancreas, promoting regeneration after injury. Activation of KRAS as in the KC mice promotes ADM but maintains metastatic cell fates preventing regeneration and instead promotes PanIN formation, a precursor lesion that gives rise to PDAC. We predict that restoring cilia will enable KC cells to respond to Hh and thereby promote redifferentiation and, in the case of an *in vitro* ADM assay, reduce the propensity for KC cells to form ductal cysts.

**Fig. 6.**
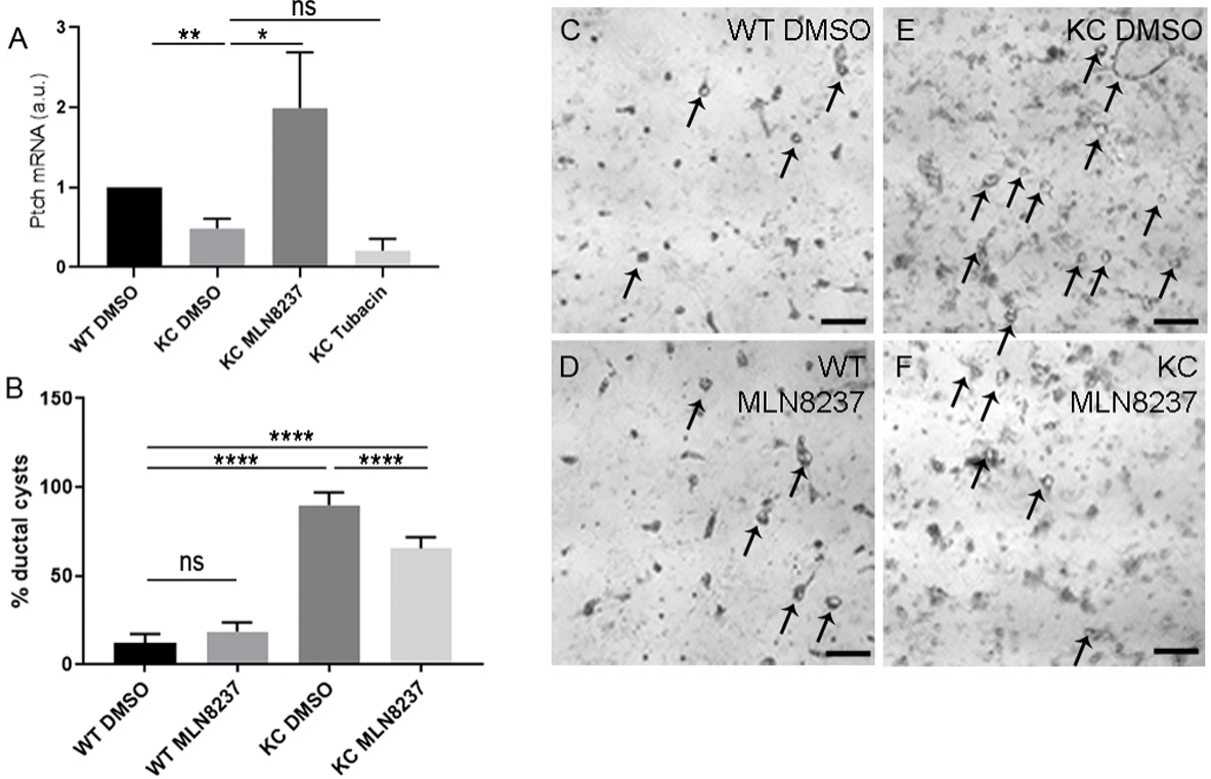
**Inhibition of AurkA restores response to Hh signalling and reduces the capacity of KC acini to undergo ADM.** (A) qPCR shows that *Ptch1* expression is significantly increased in KC ADM cells treated with MLN8237 following rescue of primary cilia compared to DMSO-treated controls (*P*=0.012, *n*=3). Tubacin treatment failed to rescue ciliogenesis or *Ptch1* expression. a.u., arbitrary units. (B) Quantification of ADM assays showing that the capacity of KC acini to form ductal cysts is reduced upon AurkA inhibition. Percentage ductal cyst formation: 12.42±1.5% in DMSO-treated WT controls, 18.6±1.6% in WT cells treated with MLN8237, 89.98±2.2% in DMSO-treated KC ADM cells, 65.39±2.1% in MLN8237-treated KC ADM cells (*n*=3 biological replicates). (C-F) Brightfield images of representative ADM assays; arrows indicate ductal cysts (*n*=3 biological replicates). Scale bars: 100 μm. Data are mean±s.e.m. ns, non-significant; *P<0.05, **P<0.01, ****P<0.0001 (two-way ANOVA).

To assess whether rescuing cilia could restore the capacity for KC ADM cells to respond to Hh signalling, we used qPCR to determine the expression levels of the Hh target gene *Ptch1*. Following treatment with MLN8237, *Ptch1* expression was significantly upregulated in KC ADM cells compared to DMSO-treated controls (Fig. 6A; *P*=0.0491, *n*=6), indicating that rescue of ciliogenesis through inhibition of the cilium disassembly pathway enables KC cells to respond to Hh signalling.

We next wanted to establish whether rescuing the ability for KC ADM cells to respond to Hh signalling could reduce their capacity to form ductal cysts. Treatment of WT ADM cells with MLN8237 had no effect on ductal cyst formation [18.6±1.6% ductal cysts formed compared to 12.42±1.5% in DMSO-treated WT controls (Fig. 6B-D; *n*=11 wells across three biological replicates)]. The number of ductal cysts formed by KC ADM cells following AurkA inhibition with MLN8237 was significantly reduced from 89.98±2.2% in DMSO-treated KC ADM cells to 65.39±2.1% in MLN8237-treated cells (*P*<0.0001) (Fig. 6B,F; *n*=10 wells across three biological replicates). This suggests that rescuing primary cilia and restoring KC ADM cells’ ability to respond to Hh signals can reduce their propensity to undergo cyst formation.

## DISCUSSION

ADM is a normal homeostatic process that, following inflammation, shuts down digestive enzyme production through dedifferentiation of acini cells to prevent further tissue damage. These progenitor-like cells then function as facultative stem cells to regenerate the pancreas; however, oncogenic transformation maintains cells in a dedifferentiated state, blocking regeneration and resulting in PanIN formation. KRAS promotes a progenitor-like cell fate, by maintaining progenitor gene expression via MAPK, PI3K, Notch, PKD1 and NF-κB signalling (Storz, 2017), and by blocking the transcriptional response to β-catenin, which drives acinar cell differentiation during development (Keefe et al., 2012; Morris et al., 2010). Here, we show that, in addition to maintaining a progenitor fate, KRAS also blocks regeneration by inhibiting ciliogenesis and thereby rendering progenitor-like cells deaf to Hh signals.

It has been reported that primary cilia are lost from low-grade PanIN lesions; therefore, we assessed the status of ciliogenesis during ADM to understand how Hh signalling is transduced following ADM in the presence of oncogenic KRAS. Using an *in vitro* system, we show that WT acini ciliate upon ADM, as previously reported *in vivo*, express Ihh and upregulate the Hh target gene *Ptch1*. We show that acini from KC mice also assemble primary cilia upon ADM; however, the number of ciliated cells is significantly reduced and, consequently, the ability of these cells to respond to Hh signals is decreased. This suggests that KRAS maintains cells in a dedifferentiated state by preventing ciliogenesis, thereby rendering these cells deaf to Hh signals.

To test our hypothesis that regeneration of progenitor-like cells is blocked in the presence of KRAS due to cilia loss and thereby inhibition of Hh signalling, we rescued cilia using an AurkA inhibitor to block the cilium disassembly pathway, a mechanism that has been shown to rescue ciliogenesis in other systems (Bangs et al., 2015). This resulted in a significant increase in primary cilia upon ADM, indicating that these cells have the capacity to assemble cilia as demonstrated by a mature basal body and presence of IFT machinery. This permitted expression of the Hh target gene *Ptch1* in KC ADM cells, showing that rescuing primary cilia can restore Hh signalling in these cells.

In our *in vitro* ADM cultures, we observe that acini from KC mice more readily form ductal cysts, as shown previously (Shi et al., 2013). This indicates that maintenance of a dedifferentiated fate through ectopic KRAS activity allows formation of more ductal cysts compared to WT cells during a 7-day culture period. Rescue of primary cilia in KC ADM cells and restoration of Hh signalling resulted in a decrease in formation of ductal cysts over 7 days, suggesting that upregulation of Hh signalling in KC ADM cells reduces the ability of KRAS to maintain a dedifferentiated fate and therefore fewer ductal cysts are able to form. Further experiments to verify the fate of progenitor-like cells were attempted; however, owing to the nature of this assay, necessitating extraction of cells from collagen, insufficient sample was obtained.

Ciliogenesis is tightly coupled with the cell cycle, in which cilia are assembled during interphase but disassemble prior to mitosis. As such, highly proliferative cells have a lower prevalence of primary cilia as more cells are undergoing mitosis. Using Ki67, we observed no difference in proliferation between WT and KC ADM cells *in vitro*, suggesting that the lack of cilia in KC cells is not due to higher proliferation and also indicating that cilia loss does not promote proliferation in these cells. We also predict that, once assembled, the cilium is stable and remains present unless the cell enters mitosis or *in vivo* dedifferentiates to an acinar cell.

Rescue of primary cilia was achieved using MLN8237 to inhibit AurkA. KRAS promotes AurkA expression downstream of MAPK via ETS2 transcription factor (Furukawa et al., 2006), and we see a significant increase in levels of total AurkA protein in KC ADM cells compared to WT. We also see an increased in phosphorylated AurkA in KC cells compared to WT, suggesting that KRAS may also promote AurkA activation through phosphorylation. Further studies will be required to understand the molecular nature of this regulation.

We have shown that rescuing primary cilia restored Hh signalling and reduced the extent of ductal cyst formation by KC ADM cells. Rescuing cilia could therefore have therapeutic benefit to enable Hh signalling following ADM promotion of regeneration while preventing the development of precursor lesions. Such a therapeutic strategy has the potential to prevent PDAC by treating individuals with chronic inflammatory pancreatic conditions such as pancreatitis, which is known to increase the risk of PDAC 16-fold (Lowenfels et al., 1993). Rescuing primary cilia may also have therapeutic benefit to patients with established disease by enabling tumour epithelial cells to regulate ectopic Hh activation through expression of negative regulators such as Ptch1 (Cervantes et al., 2010).

The most significant reason for poor prognosis for PDAC patients is our inability to detect the disease at an early stage, but even if detected early, treatment options are limited and rarely curative. Here, we address how the normal homeostatic repair process of ADM is affected by oncogenic transformation, a step that occurs prior to the development of precursor lesions. We have identified a tipping point between regeneration versus neoplastic progression and propose a mechanism by which KRAS swings the balance towards neoplasia by blocking ciliogenesis, such that cells are unable to respond to Hh and regenerate. By understanding these early initiating events, we will continue to identify potential therapeutic strategies to prevent progression to PDAC.

## MATERIALS AND METHODS

### Mouse breeding

Animal experiments were performed under a UK Home Office issued project licence and approved by the local ethical review committee. KRAS^LSL-G12D^;Pdx1Cre^+^ mice were previously described (Hingorani et al., 2003) and genotyped as per The Jackson Laboratory protocol 29,388, maintained on a C57/Bl6 background. Equal numbers of males and females were used at 5-6 weeks of age.

### ADM assay

Acinar cell 3D culture was performed as previously described (Qu and Konieczny, 2013). Briefly, pancreta from two 5- to 6-week-old WT and KC littermates were dissociated using 10 mg/ml collagenase P (Sigma-Aldrich, 11213857001) for 20 min at 37°C with agitation. The dissociated pancreta were then washed three times in Hanks’ balanced salt solution (HBSS; Gibco, 14175095) with 5% fetal bovine serum (FBS) before pipetting through a 500-µm (PluriSelect, 352360) and then 100-µm (Corning, 352360) cell sieve. Strained acinar cell clusters were then layered on HBSS+30% FBS and centrifuged at 112 ***g***. The cell pellet was resuspended in 3D culture medium: RPMI (Gibco, 21875-034) containing 50 ng/ml TGFα (Peprotech), 1% FBS, 1% penicillin/streptomycin (Gibco, 15140122), 1 µg/ml dexamethasone (Abcam, ab142419) and either 250 nM MLN8237 (SelleckChem, S1133), 5 µM Tubacin (Stratech, A4501-APE) or DMSO for untreated controls. This was mixed 1:1 with rat tail collagen (Thermo Fisher Scientific, A1048301) and plated in a 24 well plate pre-coated with collagen or a four-well glass-bottom µ-Slide (Ibidi, 80427). Culture medium was added to the wells once set and exchanged 1 and 3 days after plating. Cultures were analysed after 7 days.

### RNA extraction and qPCR

Ductal cysts were extracted from collagen using 10 mg/ml collagenase P digestion for 20 min at 37°C, washed in HBSS and centrifuged at 112 ***g*** for 5 min. RNA was extracted using a ReliaPrep RNA cell miniprep system (Promega, Z6011). Complementary DNA was synthesised using LunaScript (NEB, E3010). qPCR was performed using Luna Universal qPCR master mix (NEB, M3003) on a 7500 Fast Real-Time PCR system (Applied Biosystems). Primers used were as follows: *Ptch1*, Fwd 5′-AAAGAACTGCGGCAAGTTTTTG-3′, Rev 5′-CTTCTCCTATCTTCTGACGGGT-3′; *Gli1*, Fwd 5′-TGGACTCTCTTGACCTGGACAAC-3′, Rev 5′-GGCCCTGGGCCTCATC-3′. The annealing temperature was 38°C for 40 cycles.

### Western blot analysis

Ductal cysts were lysed in standard RIPA buffer (0.15 mM NaCl/0.05 mM Tris-HCl, pH 7.2/1% Triton X-100/1% sodium deoxycholate/0.1% SDS) with Phosphatase Inhibitor Cocktails 1 and 2 (Sigma-Aldrich, P5726 P0044) at 4°C for 30 min. Lysates were run on 4-12% Bis-Tris gels transferred onto PVDF membranes for 2 h at room temperature at 100 V. Membranes were blocked in 5% bovine serum albumin (BSA) or 5% non-fat milk in 0.1% Triton X-100/TBS buffer. Antibodies used were as follows: anti-AurkA (1:1000; BD Biosciences, 610938 IAK1), anti-pAurkA (T288) (1:100; Cell Signaling Technology, C39D8) and anti-β-actin (1:1000; ProteinTech, 60008).

### ELISA

Mouse Ihh ELISA kit (Cusabio, CBS-E16517m) was used as per the manufacturer’s instructions and read on a POLARstar Omega plate reader.

### Immunofluorescence staining

Ductal cysts were stained whole mount or after sectioning. For whole-mount staining, cysts were cultured in cover glass-bottomed wells (Ibidi), fixed in 4% paraformaldehyde (PFA) for 20 min at room temperature, permeabilized in 0.5% Triton X-100/PBS for 30 min at room temperature, washed in 100 mM glycine/PBS and blocked in 0.2%Triton X-100, 0.05% Tween20, 0.02% BSA, 10% FBS/PBS. Antibodies were diluted in blocking buffer and incubated overnight at 4°C. Wells were washed in blocking buffer and incubated with secondary antibodies plus DAPI for 3 h at room temperature, then washed in blocking buffer.

For staining on sections, collagen disks containing ductal cysts were fixed in 4% PFA for 10 min at room temperature, followed by 100% methanol at −20°C for 5 min, permeabilized in 0.5% Triton X-100/PBS for 30 min, then washed in 30% sucrose/PBS overnight at 4°C. Collagen disks were then embedded in optimal cutting temperature compound (OCT) and cryosectioned at 12 µm. Sections were washed with PBS and blocked in 1% goat serum/PBS for 30 min. Primary antibody was diluted in blocking buffer and incubated at 4°C overnight. Sections were washed four times in blocking buffer and incubated in secondary antibody for 2 h at room temperature. Slides were mounted with Prolong Gold mounting medium (Life Technologies). Antibodies used were as follows: anti-ARL13B (1:500; ProteinTech, 17711-1-AP), anti-CP110 (1:500; ProteinTech, 12780-1-AP), anti-CEP164 (1:500; ProteinTech, 22227-1-AP), anti-IFT88 (1:500; ProteinTech, 13967-1-AP), anti-acetylated α-tubulin (1:1000; Sigma-Aldrich, T6793), anti-γ-tubulin (1:1000; Sigma-Aldrich, T6557), anti-amylase (1:200; Santa Cruz Biotechnology, discontinued), anti-SOX9 (1:500; Millipore, AB5535). Alexa Fluor dye-conjugated secondary antibodies were used (Invitrogen). Imaging was performed on a Zeiss LSM 780 scanning confocal microscope or an Andor Dragonfly 200 high-speed spinning disk confocal microscope.

### Statistical analyses

Sample sizes were determined by the nature of the experiment and variability of the output. Numbers of biological replicates and cells counted for presence of cilia are provided in the text and figure legends. Data are mean±s.e.m. and two-way ANOVA was used for statistical analysis. Cilia prevalence was calculated as a percentage of interphase cells as cells in mitosis disassemble their cilium. Interphase cells were identified as cells without condensed chromosomes.

## References

[DMM044289C1] Bangs F. and Anderson K. V. (2016). Primary cilia and mammalian hedgehog signaling. *Cold Spring Harb. Perspect. Biol.* 9, a028175 10.1101/cshperspect.a028175 PMC5411695 27881449

[DMM044289C2] Bangs F. K., Schrode N., Hadjantonakis A.-K. and Anderson K. V. (2015). Lineage specificity of primary cilia in the mouse embryo. *Nat. Cell Biol.* 17, 113-122. 10.1038/ncb3091 25599390 PMC4406239

[DMM044289C3] Brune K. Abe T., Canto M., O'malley L., Klein A. P. Maitra A. Volkan Adsay N., Fishman E. K., Cameron J. L. Yeo C. J. et al. (2006). Multifocal neoplastic precursor lesions associated with lobular atrophy of the pancreas in patients having a strong family history of pancreatic cancer. *Am. J. Surg. Pathol.* 30, 1067-1076. 16931950 PMC2746409

[DMM044289C4] Cano D. A., Murcia N. S., Pazour G. J. and Hebrok M. (2004). Orpk mouse model of polycystic kidney disease reveals essential role of primary cilia in pancreatic tissue organization. *Development* 131, 3457-3467. 10.1242/dev.01189 15226261

[DMM044289C5] Carrière C. Young A. L., Gunn J. R., Longnecker D. S. and Korc M. (2009). Acute pancreatitis markedly accelerates pancreatic cancer progression in mice expressing oncogenic Kras. *Biochem. Biophys. Res. Commun.* 382, 561-565. 10.1016/j.bbrc.2009.03.068 19292977 PMC2927854

[DMM044289C6] Cervantes S., Lau J., Cano D. A., Borromeo-Austin C. and Hebrok M. (2010). Primary cilia regulate Gli/Hedgehog activation in pancreas. *Proc. Natl. Acad. Sci. USA* 107, 10109-10114. 10.1073/pnas.0909900107 20479231 PMC2890485

[DMM044289C7] Collins M. A., Yan W., Sebolt-Leopold J. S. and Pasca Di Magliano M. (2014). MAPK signaling is required for dedifferentiation of acinar cells and development of pancreatic intraepithelial neoplasia in mice. *Gastroenterology* 146, 822-834.e7. 10.1053/j.gastro.2013.11.052 24315826 PMC4037403

[DMM044289C8] De La O J.-P., Emerson L. L., Goodman J. L., Froebe S. C., Illum B. E., Curtis A. B. and Murtaugh L. C. (2008). Notch and Kras reprogram pancreatic acinar cells to ductal intraepithelial neoplasia. *Proc. Natl. Acad. Sci. USA* 105, 18907-18912. 10.1073/pnas.0810111105 19028876 PMC2585942

[DMM044289C9] Desai B. M., Oliver-Krasinski J., De Leon D. D., Farzad C., Hong N., Leach S. D. and Stoffers D. A. (2007). Preexisting pancreatic acinar cells contribute to acinar cell, but not islet beta cell, regeneration. *J. Clin. Invest.* 117, 971-977. 10.1172/JCI29988 17404620 PMC1838936

[DMM044289C10] Fendrich V., Esni F., Garay M. V. R., Feldmann G., Habbe N., Jensen J. N., Dor Y., Stoffers D., Jensen J., Leach S. D. et al. (2008). Hedgehog signaling is required for effective regeneration of exocrine pancreas. *Gastroenterology* 135, 621 10.1053/j.gastro.2008.04.011 18515092 PMC2666349

[DMM044289C11] Furukawa T., Kanai N., Shiwaku H. O., Soga N., Uehara A. and Horii A. (2006). AURKA is one of the downstream targets of MAPK1/ERK2 in pancreatic cancer. *Oncogene* 25, 4831 10.1038/sj.onc.1209494 16532023

[DMM044289C12] Graser S. Stierhof Y.-D., Lavoie S. B., Gassner O. S., Lamla S., Le Clech M. and Nigg E. A. (2007). Cep164, a novel centriole appendage protein required for primary cilium formation. *J. Cell Biol.* 179, 321-330. 10.1083/jcb.200707181 17954613 PMC2064767

[DMM044289C13] Grippo P. J. Nowlin P. S., Demeure M. J., Longnecker D. S. and Sandgren E. P. (2003). Preinvasive pancreatic neoplasia of ductal phenotype induced by acinar cell targeting of mutant Kras in transgenic mice. *Cancer Res.* 63, 2016-2019. 12727811

[DMM044289C14] Guerra C., Schuhmacher A. J., Cañamero M., Grippo P. J., Verdaguer L., Pérez-Gallego L., Dubus P., Sandgren E. P. and Barbacid M. (2007). Chronic pancreatitis is essential for induction of pancreatic ductal adenocarcinoma by K-Ras oncogenes in adult mice. *Cancer Cell* 11, 291-302. 10.1016/j.ccr.2007.01.012 17349585

[DMM044289C15] Habbe N. Shi G., Meguid R. A., Fendrich V., Esni F., Chen H., Feldmann G., Stoffers D. A., Konieczny S. F., Leach S. D. et al. (2008). Spontaneous induction of murine pancreatic intraepithelial neoplasia (mPanIN) by acinar cell targeting of oncogenic Kras in adult mice. *Proc. Natl. Acad. Sci. USA* 105, 18913-18918. 10.1073/pnas.0810097105 19028870 PMC2596215

[DMM044289C16] Hingorani S. R., Petricoin E. F., Maitra A., Rajapakse V., King C., Jacobetz M. A., Ross S., Conrads T. P., Veenstra T. D., Hitt B. A. et al. (2003). Preinvasive and invasive ductal pancreatic cancer and its early detection in the mouse. *Cancer Cell*, 4, 437 10.1016/S1535-6108(03)00309-X 14706336

[DMM044289C17] Hu S., Lu Y., Orr B., Godek K., Mustachio L. M., Kawakami M., Sekula D., Compton D. A., Freemantle S. and Dmitrovsky E. (2015). Specific CP110 Phosphorylation Sites Mediate Anaphase Catastrophe after CDK2 Inhibition: Evidence for Cooperation with USP33 Knockdown. *Mol. Cancer Ther.* 14, 2576-2585. 10.1158/1535-7163.MCT-15-0443 26304236 PMC4636444

[DMM044289C18] Jensen J. N., Cameron E., Garay M. V. R., Starkey T. W., Gianani R. and Jensen J. (2005). Recapitulation of elements of embryonic development in adult mouse pancreatic regeneration. *Gastroenterology* 128, 728 10.1053/j.gastro.2004.12.008 15765408

[DMM044289C19] Kayed H., Kleeff J., Keleg S., Buchler M. W. and Friess H. (2003). Distribution of Indian hedgehog and its receptors patched and smoothened in human chronic pancreatitis. *J. Endocrinol.* 178, 467-478. 10.1677/joe.0.1780467 12967338

[DMM044289C20] Kayed H. Kleeff J., Osman T., Keleg S., Büchler M. W. and Friess H. (2006). Hedgehog signaling in the normal and diseased pancreas. *Pancreas* 32, 119-129. 10.1097/01.mpa.0000202937.55460.0c 16552330

[DMM044289C21] Keefe M. D., Wang H., De La O J.-P., Khan A., Firpo M. A. and Murtaugh L. C. (2012). β-catenin is selectively required for the expansion and regeneration of mature pancreatic acinar cells in mice. *Dis. Model. Mech.* 5, 503-514. 10.1242/dmm.007799 22266944 PMC3380713

[DMM044289C22] Kobayashi T. Nakazono K., Tokuda M., Mashima Y., Dynlacht B. D. and Itoh H., (2016). HDAC2 promotes loss of primary cilia in pancreatic ductal adenocarcinoma. *EMBO Rep.* 18, 334-343. 10.15252/embr.201541922 28028031 PMC5286357

[DMM044289C23] Li D., Zhu J., Firozi P. F., Abbruzzese J. L., Evans D. B., Cleary K., Friess H. and Sen S. (2003). Overexpression of Oncogenic STK15/BTAK/Aurora A kinase in human pancreatic cancer. *Clin. Cancer Res.* 9, 991-997. 12631597

[DMM044289C24] Liou G.-Y., Döppler H., Necela B., Edenfield B., Zhang L., Dawson D. W. and Storz P. (2015). Mutant KRAS-induced expression of ICAM-1 in pancreatic acinar cells causes attraction of macrophages to expedite the formation of precancerous lesions. *Cancer Discov.* 5, 52-63. 10.1158/2159-8290.CD-14-0474 25361845 PMC4293204

[DMM044289C25] Lodh S., O'hare E. A. and Zaghloul N. A (2014). Primary cilia in pancreatic development and disease. *Birth Defects Res. C Embryo Today Rev.* 102, 139-158. 10.1002/bdrc.21063 PMC4213238 24864023

[DMM044289C26] Lowenfels A. B., Maisonneuve P., Cavallini G., Ammann R. W., Lankisch P. G., Andersen J. R., Dimagno E. P., Andren-Sandberg A. and Domellof L. ., (1993). Pancreatitis and the Risk of Pancreatic Cancer. *N. Engl. J. Med.* 328, 1433 10.1056/NEJM199305203282001 8479461

[DMM044289C27] Mcguigan A. Kelly P., Turkington R. C., Jones C., Coleman H. G. and Mccain R. S. (2018). Pancreatic cancer: A review of clinical diagnosis, epidemiology, treatment and outcomes. *World J. Gastroenterol.* 24, 4846-4861. 10.3748/wjg.v24.i43.4846 30487695 PMC6250924

[DMM044289C28] Means A. L. Meszoely I. M., Suzuki K., Miyamoto Y., Rustgi A. K., Coffey R. J. Jr., Wright C. V., Stoffers D. A. and Leach S. D. (2005). Pancreatic epithelial plasticity mediated by acinar cell transdifferentiation and generation of nestin-positive intermediates. *Development* 132, 3767-3776. 10.1242/dev.01925 16020518

[DMM044289C29] Miyatsuka T., Kaneto H., Toshihiko S., Matsuoka T., Yamamoto K., Kato K., Nakamura Y., Akira S., Takeda K., Kajimoto Y. et al. (2006). Persistent expression of PDX-1 in the pancreas causes acinar-to-ductal metaplasia through Stat3 activation. *Genes Dev.* 20, 1435-1440. 10.1101/gad.1412806 16751181 PMC1475756

[DMM044289C30] Morris J. P., Cano D. A., Sekine S., Wang S. C. and Hebrok M. (2010). Beta-catenin blocks Kras-dependent reprogramming of acini into pancreatic cancer precursor lesions in mice. *J. Clin. Invest.* 120, 508-520. 10.1172/JCI40045 20071774 PMC2810083

[DMM044289C31] Nielsen S. K. Møllgård K., Clement C. A., Veland I. R., Awan A., Yoder B. K., Novak I. and Christensen S. T. (2008). Characterization of primary cilia and Hedgehog signaling during development of the human pancreas and in human pancreatic duct cancer cell lines. *Dev. Dyn.* 237, 2039-2052. 10.1002/dvdy.21610 18629868

[DMM044289C32] Parsa I. Longnecker D. S., Scarpelli D. G., Pour P., Reddy J. K. and Lefkowitz M. (1985). Ductal metaplasia of human exocrine pancreas and its association with carcinoma. *Cancer Res.* 45, 1285-1290. 2982487

[DMM044289C33] Plotnikova O. V., Golemis E. A. and Pugacheva E. N. (2008). Cell cycle-dependent ciliogenesis and cancer. *Cancer Res.* 68, 2058-2061. 10.1158/0008-5472.CAN-07-5838 18381407 PMC2546565

[DMM044289C34] Pugacheva E. N., Jablonski S. A., Hartman T. R., Henske E. P. and Golemis E. A. (2007). HEF1-dependent Aurora A activation induces disassembly of the primary cilium. *Cell* 129, 1351-1363. 10.1016/j.cell.2007.04.035 17604723 PMC2504417

[DMM044289C100] Qu C. and Konieczny S. F. (2013). Pancreatic acinar cell 3-dimensional culture. *Bio. Protoc.* 3, e930 10.21769/bioprotoc.930 PMC4957951 27453905

[DMM044289C35] Rahib L. Smith B. D., Aizenberg R., Rosenzweig A. B., Fleshman J. M. and Matrisian L. M. (2014). Projecting cancer incidence and deaths to 2030: the unexpected burden of thyroid, liver, and pancreas cancers in the United States. *Cancer Res.* 74, 2913-2921. 10.1158/0008-5472.CAN-14-0155 24840647

[DMM044289C36] Schmidt T. I. Kleylein-Sohn J., Westendorf J., Le Clech M., Lavoie S. B., Stierhof Y.-D. and Nigg E. A. ., (2009). Control of centriole length by CPAP and CP110. *Curr. Biol.* 19, 1005-1011. 10.1016/j.cub.2009.05.016 19481458

[DMM044289C37] Seeley E. S., Carriere C., Goetze T., Longnecker D. S. and Korc M. (2009). Pancreatic cancer and precursor pancreatic intraepithelial neoplasia lesions are devoid of primary cilia. *Cancer Res.* 69, 422-430. 10.1158/0008-5472.CAN-08-1290 19147554 PMC2629528

[DMM044289C38] Shi G. Direnzo D., Qu C., Barney D., Miley D. and Konieczny S. F. (2013). Maintenance of acinar cell organization is critical to preventing Kras-induced acinar-ductal metaplasia. *Oncogene* 32, 1950-1958. 10.1038/onc.2012.210 22665051 PMC3435479

[DMM044289C39] Siegel R. L., Miller K. D. and Jemal A. (2015). Cancer statistics. *CA Cancer J. Clin.* 65, 5-29. 10.3322/caac.21254 25559415

[DMM044289C40] Song S. Y., Gannon M., Washington M. K., Scoggins C. R., Meszoely I. M., Goldenring J. R., Marino C. R., Sandgren E. P., Coffey R. J., Wright C. V. E. et al. (1999). Expansion of Pdx1-expressing pancreatic epithelium and islet neogenesis in transgenic mice overexpressing transforming growth factor alpha. *Gastroenterology* 117, 1416-1426. 10.1016/S0016-5085(99)70292-1 10579983

[DMM044289C41] Storz P (2017). Acinar cell plasticity and development of pancreatic ductal adenocarcinoma. *Nat. Rev. Gastroenterol. Hepatol.* 14, 296 10.1038/nrgastro.2017.12 28270694 PMC6036907

[DMM044289C42] Tanos B. E., Yang H.-J., Soni R., Wang W.-J., Macaluso F. P., Asara J. M. and Tsou M.-F. B. (2013). Centriole distal appendages promote membrane docking, leading to cilia initiation. *Genes Dev.* 27, 163-168. 10.1101/gad.207043.112 23348840 PMC3566309

[DMM044289C43] Thayer S. P., Di Magliano M. P., Heiser P. W., Nielsen C. M., Roberts D. J., Lauwers G. Y., Qi Y. P., Gysin S., Castillo C. F.-D., Yajnik V. et al. (2003). Hedgehog is an early and late mediator of pancreatic cancer tumorigenesis. *Nature* 425, 851-856. 10.1038/nature02009 14520413 PMC3688051

[DMM044289C44] Yadav S. P. Sharma N. K., Liu C., Dong L., Li T. and Swaroop A. (2016). Centrosomal protein CP110 controls maturation of the mother centriole during cilia biogenesis. *Development* 143, 1491-1501. 10.1242/dev.130120 26965371 PMC4909859

